# Local perceptions of intermittent screening and treatment for malaria in school children on the south coast of Kenya

**DOI:** 10.1186/1475-2875-11-185

**Published:** 2012-06-08

**Authors:** George Okello, Sarah N Ndegwa, Katherine E Halliday, Kara Hanson, Simon J Brooker, Caroline Jones

**Affiliations:** 1 Social and Behavioural Research Group, Kenya Medical Research Institute-Wellcome Trust Collaborative Programme, Kilifi, Kenya; 2 Malaria Public Health & Epidemiology Group, Kenya Medical Research Institute-Wellcome Trust Collaborative Programme, Nairobi, Kenya; 3 Faculty of Infectious and Tropical Diseases, London School of Hygiene and Tropical Medicine, London, UK; 4 Faculty of Policy and Public Health, London School of Hygiene and Tropical Medicine, London, UK; 5 Centre for Tropical Medicine, Nuffield Department of Clinical Medicine, University of Oxford, Oxford, UK; 6 Department of Public Health & Primary Care, University of Oxford, Oxford, UK

## Abstract

**Background:**

The intermittent screening and treatment (IST) of school children for malaria is one possible intervention strategy that could help reduce the burden of malaria among school children. Future implementation of IST will not only depend on its efficacy and cost-effectiveness but also on its acceptability to parents of the children who receive IST, as well as those responsible for its delivery. This study was conducted alongside a cluster-randomized trial to investigate local perceptions of school-based IST among parents and other stakeholders on the Kenyan south coast.

**Methods:**

Six out of the 51 schools receiving the IST intervention were purposively sampled, based on the prevalence of *Plasmodium* infection, to participate in the qualitative study. Twenty-two focus group discussions and 17 in-depth interviews were conducted with parents and other key stakeholders involved in the implementation of school health programmes in the district. Data analysis was guided by the framework analysis method.

**Results:**

High knowledge of the burden of clinical malaria on school children, the perceived benefits of preventing clinical disease through IST and previous positive experiences and interactions with other school health programmes facilitated the acceptability of IST. However, lack of understanding of the consequences of asymptomatic parasitaemia for apparently healthy school children could potentially contribute to non-adherence to treatment, and use of alternative anti-malarial drugs with simpler regimens was generally preferred. The general consensus of stakeholders was that health workers were best placed to undertake the screening and provide treatment, and although teachers’ involvement in the programme is critical, most participants were opposed to teachers taking finger-prick blood samples from children. There was also a strong demand for the distribution of mosquito nets to augment IST.

**Conclusion:**

School-based malaria control through IST was acceptable to most parents and other stakeholders, but careful consideration of the various roles of teachers, community health workers, and health workers, and the use of anti-malarial drugs with simpler regimens are critical to its future implementation.

## Background

The importance of user and provider perceptions to the successful implementation and use of public health interventions is widely recognized [1,2]. Over recent years, socio-behavioural research has increasingly been used to inform the development and implementation of appropriate interventions for the prevention and control of a variety of infectious diseases, including malaria. For example, a number of studies have investigated the acceptability of interventions to prevent malaria such as: the use of insecticide-treated bed nets [3,4]; intermittent preventive treatment in infants [5-8]; indoor residual spraying [9] and, more recently, intermittent preventive treatment *versus* intermittent screening and treatment in pregnancy [10]. Other studies have investigated the acceptability of new anti-malarial drugs and dosage regimes [11] and strategies for treatment delivery [12-14]. The results from these various acceptability studies have demonstrated that intervention acceptability is influenced by a range of factors including: perceptions of the burden of disease and disease aetiology; perceptions of the safety and effectiveness of the intervention; perceptions of the benefits of the intervention; individual, social and cultural factors; and structural and system factors [3-14].

**Figure 1:**
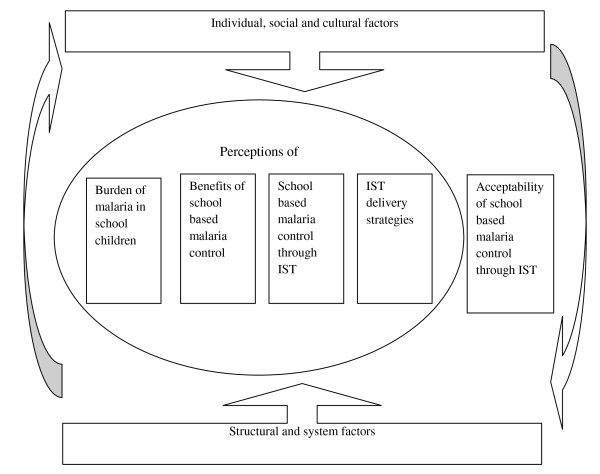
Conceptual framework.

To date, much of the focus on the development and implementation of interventions for malaria prevention and control has been on those population groups who are perceived to be at greatest risk from the disease, namely pregnant women and children under five years [15]. However, malaria remains an important cause of mortality and morbidity in school children and may have profound consequences for their learning and educational achievements [16-19]. There has been growing appreciation by many African governments of the importance of child health for educational achievement [20], and an increasing number of low-income countries now use their schools as platforms for delivering simple, safe and cost-effective health and nutrition interventions, such as de-worming [21]. However, while there is growing awareness of the importance of reducing the burden of malaria in school children and political support for school-based malaria control [21], there is still limited evidence to guide the formulation of policy [22,23]. A cluster-randomized trial (CRT) in Kenya is currently investigating the impact on burden of disease and educational outcomes of intermittent screening and treatment (IST) for malaria in school children [24]. As with other malaria control interventions, the programmatic effectiveness of this intervention in school-based malaria control will depend not only on its effectiveness and cost-effectiveness but also on its acceptability to key stakeholders such as parents, teachers and local implementers. To date, few reliable data on the acceptability of school-based treatment programmes are available from sub-Saharan Africa. There is some evidence on the acceptability of school-based de-worming [25] and school-based administration of medication for malaria treatment [26-28]. However, IST in schools is different from these programmes in that it involves parasitological diagnosis before administering treatment. In addition, in contrast to malaria treatment programmes, IST for malaria potentially involves the selective administration of medication to apparently ‘healthy’ individuals. Should IST for malaria prove to be efficacious in reducing the health and educational burden of malaria among school children then it is important to understand the local perceptions of the intervention, and its acceptability to the frontline providers and recipients of the intervention. Such information is central to future policy development and decisions about appropriate strategies for intervention implementation and delivery. This study aimed to investigate the local perceptions and acceptability of IST for school children in two coastal districts in Kenya. The study was guided by a conceptual framework (Figure 1) developed from the findings of previous malaria intervention acceptability studies [3-11]. The framework illustrates how individual, social and cultural factors as well as structural and system context (supply chain, human resources, health infrastructure and relationships between the ministry of education and ministry of health) surround and influence both perceptions of disease and the acceptability of the process adopted for its control.

## Methods

The study is part of a multi-sectoral impact evaluation of school-based IST on the south coast of Kenya undertaken between January 2010 and May 2012. Details of the study design and intervention characteristics have been described elsewhere [24]. In brief, a CRT is evaluating the impact of two interventions among randomly selected children from classes 1 and 5 in 101 primary schools: (i) IST of malaria in schools by public health workers and (ii) training workshops and support for teachers to promote explicit and systematic literacy instruction. The primary outcomes are educational achievement and anaemia, and secondary outcomes include malaria parasitaemia, school attendance, sustained attention and other cognition abilities. The focus of this qualitative study was the IST intervention.

### Study setting and population

The impact evaluation is being carried out in Kwale and Msambweni districts, Coast Province, Kenya. The population in the area consists of a mix of ethnic groups, with the largest groups being Digos, Kambas, and Durumas, and is predominantly Muslim but with a sizeable Christian population. The main economic activities are subsistence farming, fishing and tourism. Malaria transmission is moderate and perennial, with two seasonal peaks occurring during the long rains between April and June and the short rains between October and November [24]. The geography and epidemiology of malaria in the area have been described in detail elsewhere [29]. The two districts are some of the poorest and least developed districts in Kenya and consistently perform poorly in the national school examinations [24].

### The IST intervention

**Figure 2:**
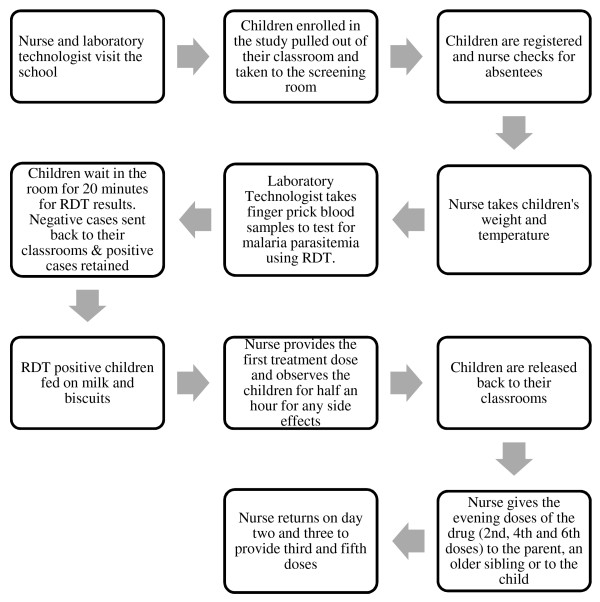
The steps involved in intermittent screening and treatment in schools.

Between February 2010 and November 2011, five rounds of school-based screening and treatment for malaria were undertaken in the 51 malaria intervention schools. The screening process is summarized in Figure 2. Mobile health teams, comprising laboratory technicians and nurses from local health facilities, visited schools each school term. Laboratory technicians took finger-prick blood samples to test for the presence of malaria parasites using ParaCheck-*Pf* malaria rapid diagnostic test (RDT) (Orchid Biomedical Systems, Goa, India), which is able to detect *Plasmodium falciparum* and other (unspecified) *Plasmodium* species*.* Children (with or without malaria symptoms) found to be RDT-positive were treated with artemether-lumefantrine, AL (Coartem®, Novartis), an artemisinin-based combination therapy. A nurse administered and directly observed the first treatment dose in the school and returned on the subsequent two days to provide the third and the fifth doses, respectively, and to monitor any side effects. The second, the fourth and the sixth doses of the drug were given to the parent if they were present in school, or to an older sibling for children in class 1. As a last resort, the drugs were given to the child to take at home along with a note to their parents explaining that they were found to be infected and instructions on taking the drugs.

### Sampling and data collection

For the acceptability study, six malaria intervention schools were purposively selected on the basis of the prevalence of *P. falciparum* infection, as determined in the 2012 baseline survey [29]. Two schools each with the highest, medium and lowest prevalence were chosen. The rationale for selecting schools with varying levels of infection prevalence was to allow for a range of responses from participants in areas of different malaria transmission intensities. Three of the six selected schools were located within a radius of 10 km of Ukunda town, where the project office is located; the other three schools were remote rural schools. Data were collected through in-depth interviews (IDIs) and focus group discussions (FGDs).

### Focus group discussions

Parents were recruited into the study with the help of village elders and school management committee leaders. They were provided with a list of names of those parents whose children were enrolled in the study and were asked to identify and approach those who came from nearby villages with information about the qualitative study and invite them to attend the FGDs. In total, 12 FGDs were conducted with parents of children enrolled in the study, two from each school. Separate FGDs were conducted with teachers (five), health workers working for the trial (one), and community health workers (four). FGDs were of mixed gender and had between five and 12 participants. FGDs were moderated by a team of two trained field workers fluent in the local language working under the supervision of the lead investigator (GO). They were provided with a pre-tested flexible topic guide to direct the discussions. Discussion topics included: perceptions of the problem of malaria in school children, malaria testing and treatment, knowledge and experiences with IST in school children, perceptions of IST delivered by teachers, community health workers (CHWs) and health workers and opinions on school health programmes. Field workers carried a sample RDT to all the FGDs and used it to explain the procedure for malaria testing. At the end of each day of field work, the lead investigator met with the two field workers to discuss emerging themes and issues that required further probing in subsequent FGDs.

### In-depth interviews

A total of 17 in-depth interviews (IDIs) were conducted with head teachers of the selected schools and members of the district school health coordinating committee, comprising representatives from both the ministries of education and health responsible for the implementation of school health programmes locally. Participants were initially contacted by telephone to identify a suitable date and time for the interview. Interviews were conducted, usually in participants’ offices, by either the lead investigator or the senior social scientist (CJ). Interview topics included: participants’ experiences of implementing school health programmes in the districts, knowledge and perceptions of school-based health programmes and IST, and opinions on options for delivering IST in schools.

### Data analysis

FGDs and IDIs were conducted in either Kiswahili, the language predominantly spoken along the Kenyan Coast, or in English. Interviews were recorded, transcribed and translated (where necessary). For quality control, all transcripts were reviewed by the lead investigator (GO) and a portion of the interviews were back-translated. Interview transcripts were managed using Nvivo 8 (QSR International, Doncaster, Australia). Data from the different sources were compared and contrasted to ensure that full ranges of views were captured in the analysis. Interpretation of the data was informed by experiences during implementation of the trial as well as from the analysis of the qualitative data. The conceptual framework (Figure 1) was used to inform a framework for data coding and further analysis. The focus of the current analysis is on local perceptions and the acceptability of IST for malaria in school children. A separate paper will discuss the implementability of school-based malaria control in schools through IST.

### Ethical approval and consent procedures

Ethical approval for the main trial was provided by the Kenya Medical Research Institute (KEMRI) and National Ethics Review Committee (ERC) (SSC No. 1543), the London School of Hygiene and Tropical Medicine Ethics Committee (5503), and the Harvard University Committee on the use of Human Subjects in Research (F17578-101). Supplementary approval for the FGDs and IDIs was received from the KEMRI and national ERC. An information sheet explaining the purpose of the study was read out to participants at the beginning of the IDI’s and FGD’s. Verbal and written informed consent was sought from all participants to take part in the in the FGDs and IDIs, respectively, and for the interview to be recorded. Additional permission was sought from the ministries of education and health both at the national and the local level. Digital recordings of interviews and transcripts were stored in password-protected computers accessible only by project staff. All the names of participants and places were removed from the transcripts and replaced by unique identifiers.

## Results

### Perception of school health programmes

The majority of parents, teachers and educational officials across all the IDIs and FGDs felt that schools were a practical entry point for health interventions that target children of school-attending age because the majority of them were in schools.

"I think the school is the entry point for this (school health programmes) simply because all the children are mostly found in the school. (Head teacher, IDI)"

A few of the education officials perceived that targeting health interventions through schools was also an effective way of reaching the rest of the population especially with health messages. This view was supported by some health workers who reported that they had used schools to pass information on disease prevention to the rest of the community. A minority of the health managers, education officials and parents observed that school children faced various health problems, which affected their education but many were unwilling to access health care due to low trust of health workers and unfamiliarity with the health system. These managers perceived that school health programmes could play an important role in addressing some of these health problems. Parents, educational officials and health managers reported that a number of school health programmes, such as de-worming and treatment for bilharzias and lymphatic filariasis, had been implemented in the area by the government. Most of these programmes were implemented in schools by teachers with the support of health workers, and were considered by parents, teachers, education officials and health managers to have been generally successful.

"I would say they (school health programmes) were successful because our children have benefitted because they now have better health. (Parent, FGD)"

However, a few teachers reported that they had encountered some challenges during the de-worming programme where some children refused to take their drugs because they suspected that they were meant for birth control.

"There are some people who discourage others that when you are given these drugs and you swallow them you are taking family planning tablets…So when you relate to that issue you find that most girls become fearful…they get worried… “I am a girl and I am being given these drugs… Why should I take them and I want to become a mother when I finish school?” So some have that fear although I talk to them and encourage them and tell them that those drugs are not for family planning. (Head teacher, IDI)"

In some isolated cases, teachers reported that parents had withdrawn their children from schools during the exercise for the same reasons. Health managers and education officials also complained that the de-worming programmes had been underfunded by the national government which made it difficult for them to effectively monitor and supervise the delivery of the programme in schools.

### Perceptions of the burden of malaria in school children

While the data was collected across different malaria transmission settings, it became clear that there was no difference on perceptions of the burden of malaria on school children and the need for control across these transmission settings.

There was a general agreement across all the transmission settings and groups that pregnant women and preschool children bore the greatest burden of malaria and that most interventions aimed at malaria control had targeted these population groups. However, the consequence of the disease on school children was also recognized by most participants in this study. The majority of participants recognized that clinical malaria was likely to result in absenteeism from school and subsequently loss in learning days, which had an implication for overall education attainment of school children. Similarly, children suffering from clinical malaria were more likely to have problems with concentration in class, which would further affect their academic performance.

"Malaria affects them while in school and I am sure they lose out on learning; they may sleep in class due to malaria. It is a disease that is harmful. (Parent, FGD)"

"It affects school performance because if the child is always absent learning cannot take place effectively. (Teacher, IDI)"

In addition, a few parents, teachers and CHWs perceived that children risked developing cerebral malaria if they suffered clinical attacks. In one FGD, parents also acknowledged the economic burden of providing treatment for their children if they fell sick with malaria. In one school, teachers reported that a child had died of anaemia after suffering from clinical malaria.

While the clinical burden of malaria on school children was apparent to most participants, only a few parents, teachers and health workers recognized that school children could suffer from asymptomatic parasitaemia which might also affect their academic performance. Some of these parents perceived that asymptomatic parasitaemia was less of a problem for school children and most of them recovered from it naturally.

"If you test the whole school right now, you will find that almost 90% of the whole school have malaria parasites. Interestingly it doesn’t affect them that much. They will just recover on their own. It keeps on coming and going. (Parent, FGD)"

However, one health manager and a teacher did recognize that asymptomatic parasitaemia was likely to lower children’s attention in class, which eventually would affect their learning abilities.

"As the malaria parasites tend to multiply you definitely cannot say that the child will be at the same learning pace with another healthy child who is learning at the same time. Definitely this one who is incubating will have problems in keeping up with the pace. (Health manager, IDI)"

### Perceptions of school-based malaria control through IST

School-based malaria control through IST was seen as a benefit to school children in a number of different ways by the various stakeholders who took part in this study. In one of the FGDs, CHWs observed that testing and treating school children for malaria would help in the early detection of asymptomatic malaria, which, in turn, would reduce its risk of progressing to clinical disease. In one of the schools, parents were very positive about the intervention because they perceived that it had reduced the risks of clinical malaria and absenteeism from school, a view that was supported by other parents, teachers and educational officials.

"I don’t have any (concerns with IST) because if my child will be tested and found to have malaria and is given medication and so they attend school well and performance improves what complains would I then have? (Parent, FGD)"

"We actually appreciate because they came and told us there is malaria and maybe it causes the children not to perform because they are always absent from the school due to malaria. If that is taken care of then that will improve (their) performance because they will always attend classes, absenteeism cases won’t be high. (Head teacher, IDI)"

Some parents and health managers also observed that screening children and targeting treatment to only those found with *Plasmodium* infection would help in reducing the risk of development of drug resistance associated with presumptive treatment which was reported to be a common problem in the area. In addition to this, a few of the health managers perceived that IST would contribute to other malaria control efforts by reducing the reservoir of transmission in the wider community.

"I think this one is quite focused because you have actually tested someone and seen parasites so you know exactly what you are dealing with. And you see this will also have the potential impact of removing the hosts because you see those with the parasites will not have the symptoms…so you are reducing transmission as well. (Health manager, IDI)"

### Concerns with school-based malaria control through IST

Despite the positive perceptions of IST in schools as a strategy for addressing the burden of malaria in school children, it was not considered to be the only approach for school-based malaria control. Some teachers, educational officials, parents, health workers and health managers argued that even if children were treated for malaria in schools, they were still exposed to re-infections at home because most of them lacked insecticide treated bed nets (ITNs). These participants recognized the value of ITNs in malaria prevention but reported that most ITNs distribution programmes had mainly targeted pregnant women and preschool children, leaving parents with the burden of having to provide ITNs to their children. In addition to IST, they suggested that school children be provided with ITNs to protect them from the bites of mosquitoes.

"Respondent 1: And I also thought that if you are testing these children and you find them with malaria even if you are treating them why are you not giving them nets?"

"Respondent 2: Because you might treat a child today and at night he sleeps without a net so the same mosquito will bite him. (Teachers, FGD)"

After the fifth round of IST, health workers working for the trial also reported that some parents were concerned with the frequency with which their children were tested and perceived that an alternative intervention that did not involve taking blood from school children would have been better than IST. This concern did not come up during discussions with parents perhaps because at the time that all, except one of the parents’ FGDs were conducted, only the first round of screening and treatment had been undertaken. However, in one FGD with parents conducted after the fifth round of testing and treatment, parents did not mention concerns about frequency of testing and perceived that taking finger-prick blood samples from school children did not involve any significant risks as long as it was done by the right people and all the safety procedures were adhered to.

"Respondent 1: How can pricking a child’s finger be a problem? What will you be doing to him?"

"Respondent 2: In fact the way I see it from what you demonstrated and what is done in schools, I think this is less risky. I don’t see how this is going to be a problem. (Parents, FGD)"

Non-adherence to treatment was also cited by health workers who implemented this intervention as well as by some parents as a possible challenge in implementing IST in schools. In the FGD associated with one school, a few parents reported that their children had informed them that they were tested for malaria in school and that they had been treated, but they had not brought the remaining doses of the drug home. These parents suggested that their children may have thrown away the drugs, either because they feared taking them or because they believed that they were not suffering from malaria. However, it is also possible that the children perceived that the testing they had undergone was some form of treatment (which they reported to their parents) and they may not have been given any drugs at school. Health workers blamed non-adherence on the complex dosage requirements of AL, which made it difficult to monitor adherence and a lack of understanding by parents and children of why they were put on treatment yet they were not sick. These health workers were of the view that a single-dose treatment may have been more effective for use in IST than AL.

"The child will just be playing then all over sudden you prick the child and you say that the child has malaria. Then you give them drugs. Take these ones now and then again in the evening you give them more drugs. So they will be wondering why you have given the child malaria drugs yet he is not sick! He has not complained of headache or anything. So the ones we give them in school they can take but the ones they are supposed to take at home if the mother is not enlightened will tell (the child)‘do not to take the drugs. What are the drugs for?’ (Health worker, FGD)"

In one FGD, a CHW working for the trial reported that a parent had used her child’s medication, given to him after testing in school, to treat another sibling who was perceived to be sicker. CHWs argued that non-adherence to treatment was a general problem in the community and was not unique to school children, a view that was supported by the majority of parents who, despite their general knowledge of the dangers of non-adherence, still reported that most of them stopped their treatment once they felt better or saved the drugs for future use.

"Like me, I was given (drugs) by the doctor but when I started feeling better with no headache, no fever, I stopped (taking the drugs) so that means we do not take the dose as should be done. We have to get a way to ensure that the dose is completed because once you feel better you stop the dose yet the sickness is still in your body. (Parent, FGD)"

Some parents blamed the non-adherence reported in this study on the influence of rumours about devil worshipping and covert HIV testing which were prevalent in the community at the beginning of the study. These rumours may have been reinforced by trial procedures such as the randomization process, the requirement to provide written informed consent, and blood sample taking. There were concerns in the general community about why the intervention only targeted specific schools, classes and children yet all children in the area were at risk of malaria. Parents also questioned why they were required to provide written informed consent for their children to participate in the intervention yet the study had been approved by the government and was run by a government research institution. There were also anecdotal reports that some parents encouraged their children not to take their drugs because of suspicions that they were given by devil worshippers. This issue was also raised in discussions with parents.

"I don’t think it is the child’s mistake (not to take the drugs). I can say it’s the parent’s mistake. Because they say these are not real drugs. They say they are devils. (Parent, FGD)"

Some parents were also convinced that blood taken from their children would be sold or be used to test HIV.

"When you started off there were many rumours circulating that you were taking the blood to sell it or to go test for HIV. (Parent, FGD)"

Such rumours created fear in school children, and health workers reported that in some schools a number of children ran away as soon as health workers arrived in school to conduct the exercise. However, parents and other stakeholders, such as education officials and teachers, were of the opinion that these concerns were less likely to be a problem under routine implementation in the absence of trial characteristics such as randomization and requirement for informed consent process. Some participants dismissed these rumours and argued that for every new intervention, there would always be some suspicions regarding it, which faded away with time as people interacted with the intervention more. In this study for instance, rumours led to initial refusals to participate and withdrawals from the study but following further community sensitization many of these children were re-enrolled. It was suggested that implementation of IST in schools be preceded by proper community sensitization to address people’s fears and concerns about it.

"I think there is need for community sensitization. You see what you were doing was done as a research project but when this is implemented by the government, there will be no issues of selecting children or requiring them to sign up. You just need to sensitize people to make them aware of the reasons for undertaking the project. (Parents, FGD)"

### Perceptions of delivery models for IST

Participants’ views were also sought on three possible delivery strategies for implementing IST in schools. These strategies included using health workers, or community health workers or teachers (Table 1) to test and treat children in schools. Results from both IDIs and FGDs with the different participants showed that there were few concerns about who delivered treatment to school children, but there were some concerns about who took blood samples from school children, whether they had the capacity to do this, and the practicality of implementing such a strategy.

**Table 1:** Possible scenarios of delivering intermittent screening and treatment to schoolchildren in schools

**Scenario A**	**Scenario B**	**Scenario C**
Health workers from local health facilities visit the school to screen and treat children. First dose given in school and remaining doses taken at home.	Teachers are selected from the school and trained on how to test and treat children (with or without health worker supervision). First dose given in school and remaining doses taken at home.	Trained CHWs visit the school to screen and treat children. First dose of the drug given in school and remaining doses taken at home.

### Perceptions of health workers as delivery agents

There was general agreement among the majority of teachers, parents and educational officials that health workers were probably the most appropriate people to deliver the IST intervention in schools because it was part of their professional roles and responsibilities. Teachers and educational officials perceived that parents and school children were more likely to be comfortable if the intervention was delivered by qualified health workers. Many parents agreed with the proposal to use health workers to deliver the intervention because they perceived that they had the knowledge and the experience to deliver the intervention safely and were better prepared to deal with any eventualities, such as side effects if they occurred.

"In my opinion I would say that the person who is already trained and has all the experience is the best person to do the testing and treatment. (Parent, FGD)"

"Of course that (screening and testing) should be done by the expert that is the clinical officers or the nurses from the health centres… (Education official, IDI)"

Nevertheless, a minority of parents in one FGD observed that children were likely run to away from health workers because they were less familiar with them and would possibly associate them with rumours about blood stealing and devil worshipping common in the community. In the same FGD, parents also perceived that health workers were likely to sell drugs meant for the intervention, as was the case in public health facilities.

Health workers working for the trial agreed that it was better to have health workers deliver the intervention in schools because it was within their professional roles and responsibilities. They were, however, concerned that the screening and treatment process in schools involved a significant amount of work which required more than two health workers per school and, because of understaffing in public health facilities, this was likely to be a challenge.

"If we have enough staff that (IST) is very easy work to do because that is under school health. The shortage of nurses now is the only problem we have. It would have been very easy. (Health worker, FGD)"

This view was supported by health managers at the district level and some CHWs who also argued that involving health workers from local public health facilities would affect the delivery of routine health care services in these facilities.

"You know a nurse is employed to provide certain services in the health facility. So if the nurse leaves to go and provide this intervention in schools the other part (normal service provision) will suffer. (CHW, FGD)"

On the other hand, one health manager at the district level suggested that if screening was done at specific time points, health workers were still in a position to deliver it.

### Perception of CHWs as delivery agents

There were mixed responses regarding the appropriateness of using CHWs to screen and treat school children. Both health workers working for the trial and health managers at the district level appreciated the role of CHWs in health promotion in the community, but pointed out that, to date, they had not been involved in the provision of interventions that involved administering injections or taking blood samples from the population. While a few of the health workers and health managers were of the view that CHWs could be used to deliver the IST intervention in schools if they were trained and supervised, the majority of them were sceptical about their use to test and treat school children. They were aware that some of the CHWs were illiterate and they perceived that this would make it difficult to train them on how to deliver the IST intervention in schools. In addition, they suggested that some parents would not trust in the ability of CHWs to screen and treat their children because they knew they were not qualified health professionals. One health manager at the district level also argued that such a strategy would turn CHWs into ‘small doctors’, which was already a problem in the community.

"It may be a challenge because you see nowadays parents know their rights. They know who is supposed to do this and who is supposed to do that. So they might refuse the services because they are used to them (CHWs). They are people they live with… and then today you are coming to test them…where did you train? They see them weigh their children but when it comes to treatment it may be difficult. (Health worker, FGD)"

Parents, teachers, and educational officials who supported the use of CHWs to deliver the intervention in schools did so from the point of view that they were more likely to be trusted by school children and parents because these were community members who were familiar to them and they already recognized their work. Some parents for instance argued that CHWs were already being used to conduct community based HIV testing and filariasis treatment in schools and could, therefore, be trained to test and treat school children for malaria and to monitor adherence to treatment in the community.

"I don’t think this is going to be a problem because you see these people have also been involved in giving filariasis drugs in schools. (Parent, FGD)"

"There is no problem as long as they (CHWs) understand what they are doing…and they will handle the children properly. We have no problem. (Teacher, FGD)"

However, not all teachers, parents and educational officials were comfortable with the proposal to use CHWs to deliver the IST intervention in schools. Like health workers, some teachers and parents were concerned that some CHWs were illiterate and as such were less likely to understand the intervention and children would also doubt their capacity to screen and treat them.

"With their (low) education level pupils will doubt (them) because they know those people (CHWs) are working through experience and are not experts. They have it in them that the community workers are not experts so for them taking their blood and testing, they will doubt the result…how can this one tell me I have malaria and he’s just a class eight leaver? (Teacher, IDI)"

In two separate FGDs from the same school, parents repeated the allegation also levelled against health workers that CHWs were likely to sell the drugs meant for the IST intervention. These concerns, however, were dismissed by parents in another school who argued that AL was issued free of charge in government health facilities and as such there was no way that health workers or CHWs could sell them. In one of the schools, some teachers were of the view that school children were more likely to refuse to be tested by CHWs because of the assumption that they would test them for HIV.

In contrast to the concerns that were raised against them, most CHWs interviewed in this study were confident that with proper training they would be in a position to deliver IST in schools because most community members already acknowledged their work in the community. They were also of the view that they were likely to be trusted by parents and school children because they were familiar to them. They were aware of the risks involved in the screening and treatment process and suggested that they have some form of health worker supervision to boost, not only their own confidence, but also that of community members and school children in their ability to do the work.

"If we are trained on how to use these kits and how to administer drugs to someone who has malaria, I think it is something that we can do because the most important thing regarding any type of work is that you should be trained on how to do it and you also need to understand it. (CHW, FGD)"

They also acknowledged concerns about their perceived low level of education. However, they did not consider this to be a major barrier to their involvement in the delivery of IST in schools as they perceived that the RDT screening process was a practical process that even those with less formal education would be able to implement. Nonetheless, they did suggest that only those with a higher level of education be trained to implement IST in schools.

"(This is) something you see with your own eyes and the more you do it the more you know it. It is true there are those of us whose understanding is quite low so may not understand this thing but if you are doing it I thought you are going to select those who are doing it perfectly to be the ones to go out and deliver it in schools? You cannot use everyone because there are those who will not be in a position to do it. (CHW, FGD)"

Some of the CHWs linked the concerns raised about their low level of education to their awareness that some community members did not appreciate the work they undertook. They also pointed to the voluntary nature of their efforts and suggested that it contributed to undermining the value of the work they performed. They suggested that providing some form of compensation would help increase their status and improve acceptability if they were to be involved in the delivery of IST in schools.

### Perceptions of teachers as delivery agents

Like CHWs, there were mixed responses from participants in both IDIs and FGDs on the proposal to use teachers to deliver IST in schools. Nonetheless, there was a general agreement that regardless of who delivered the intervention in schools, the involvement of teachers was critical to the success of the process. The majority of parents, educational officials, health workers and health managers recognized that teachers were trusted by both school children and parents because of the considerable amount of time they spent with them in schools and their social positions in the community.

"I would like to suggest that the teacher be trained to do the testing and the treatment. You see it is the teacher who spends most of the time with the child and so can easily assist in addressing malaria cases in children. (Parent, FGD)"

The majority of health managers and some educational officials suggested that since RDT screening was a simple procedure, teachers if well trained and supervised could be used to deliver IST in schools.

"If they get proper induction I don’t think they will get any problem. For them it will be an honour to do that work. And I believe they will do it with a lot of enthusiasm. (Education official, IDI)"

In addition to this, a few health managers considered that using teachers to deliver the intervention would also create local ownership of the project. By contrast, this view did not come up during interviews and discussions on the proposal to use CHWs as a possible delivery strategy. Some health workers perceived that there were minimal risks involved in using teachers to conduct the exercise in schools and argued that with proper training, supervision and support of health workers, teachers would be in a position to implement the intervention in schools.

"So if the teachers can be trained on how to administer the doses and since the child will be with the teacher presumably for all the time including the three treatment days, then if the teacher is trained and there is a framework for monitoring the intervention, I would go for that. (Heath manager, IDI)"

A few of the teachers agreed that with proper training, supervision and support, they would be comfortable to deliver IST in schools. In addition, they perceived that involving them in the IST delivery process would provide them with an opportunity to expand their knowledge on how to tackle the problem of malaria not only in schools but in the wider community as well. For teachers, the importance of supervision was seen in terms of having someone to turn to if things went wrong and also to make sure that they were doing the right thing.

"There is need for supervision because a problem may occur. You may prick the child in the vein and the bleeding refuses to stop. So there is a need for somebody to supervise the teacher. (Teacher, FGD)"

However, the majority of health workers, teachers along with parents, expressed their concerns about the capacity of teachers to take blood samples from school children. They argued that teachers were trained to teach and involving them in a process that required them to take blood samples from children was unacceptable because such roles were beyond their scope and were the preserve of health professionals. Some CHWs also perceived that teachers were less likely to adhere to the guidelines for delivering the intervention. They reported this had been the case during a school-based bilharzias treatment programme where teachers reportedly failed to administer drugs to children as had been recommended and some children developed side effects from the drugs after the exercise.

"When they (children) tell you they have been treated by a teacher then we ask is the teacher supposed to teach or is the teacher a doctor? This will bring confusion. (Parent, FGD)"

Similarly, the majority of teachers and some educational officials perceived that it was unprofessional for teachers to take blood from children and that doing so would not only erode the trust that children had in them, but also expose them to risks of contracting infectious diseases. In one school, teachers saw attempts to involve them in delivering a health intervention in schools as a form of exploitation where other people’s professional roles were being handed over to them. They were aware of the risk involved in engaging in such an activity and reported that they were ill equipped to respond to them. Teachers were also concerned that their involvement in IST would increase their workload and affect their ability to discharge normal teaching duties.

"I think you will be exposing teachers to some dangers as pertaining to that because we are not experts in medicine and we are not experts in that. Although we are learned, (we may not know) the precautions to be taken. (Teacher, FGD)"

## Discussion

This study was carried out to investigate local perceptions of school-based malaria control through IST for malaria in school children. It was clear across the different transmission settings in the study area that knowledge of malaria and its consequences was high and all stakeholders recognized the importance of tackling clinical malaria among school children; there was, however, less awareness of the importance of asymptomatic malaria. The perceptions of health managers, health workers, CHWs, educational officials and teachers and parents of the burden of malaria in school children and the benefits of school-based malaria control through IST played a significant role in the positive attitudes towards IST that were found in this study. However, there was a strong demand from parents for mosquito net distribution to be undertaken as a complementary intervention to IST to prevent clinical disease.

While IST was clearly perceived to contribute to a reduction in clinical disease, few participants appear to have been aware that the principal aim of IST is the reduction of asymptomatic parasitaemia, rather than the treatment of clinical disease. At an individual level the reduction of asymptomatic parasitaemia is important since it has been linked to iron deficiency anaemia and impaired cognitive function in school children [22,30] and at a community level it has the benefit of reducing the reservoir of parasites for malaria transmission [31]. Although this lack of awareness did not appear to impact on the acceptability of the intermittent screening component of the intervention, the findings do suggest that it may affect willingness to adhere to the full treatment regime. That is, some parents were concerned that their children were put on malaria treatment when they were perceived to be healthy. In a few cases these parents encouraged their children not to take their medication and instead used the drugs to treat other sick siblings and, in other instances, children were reported to have thrown away the tablets as they did not perceive themselves to be ill. These findings suggest that, while the concept of screening and treatment for malaria is generally acceptable, adherence to treatment given to children with asymptomatic parasitaemia may be problematic. In addition, the complex six-dose regimen of AL which requires that all doses be correctly spaced and be given with food may present a major challenge in a school setting, especially if drugs are issued to children or teachers to pass to their parents without proper information on dosage and a simpler anti-malarial regimen would enhance compliance. If successful, the implementation of IST as a strategy for school-based malaria control will need to be preceded by community education to reinforce the message of the consequence of asymptomatic malaria on the health and education of school children and the importance of IST as a strategy for school-based malaria control.

The use of health workers to implement the IST interventions in schools is likely to be acceptable because this is a health intervention, which forms part of health worker roles. However, there is a serious shortage of health workers in Kenya especially in public health facilities, and health workers in these facilities are already overworked [32]. This, coupled with the cost of such a strategy estimated to be about $6.61 per child screened [33], may limit the use of health workers to implement the IST intervention in schools. Analysis shows that delivery costs can be reduced by having CHWs or teachers implement IST [33], but regardless of who implements the intervention, the support of health workers is critical to the successful implementation of the IST intervention in schools. Their involvement is particularly necessary in terms of training and supervising the delivery agents implementing the strategy in schools, in facilitating safe waste disposal, and in handling referral cases arising from schools.

Several studies have found the use of CHWs in the home management of malaria to be acceptable [13,14]. In this study, the use of CHWs to deliver the IST intervention in schools appeared to have been acceptable to some participants who trusted and respected them and perceived them to be committed to promoting the health of their communities. They were perceived to be more available and accessible than health workers or teachers because they live in the community and could therefore be used to monitor adherence to treatment at home. With proper training, supervision and support from health workers, the use of CHWs may be an acceptable alternative to the use of health workers or teachers to implement the IST intervention in schools. The main concern regarding their use to deliver IST in schools centred on perceptions that some of them are insufficiently educated to be able to understand the intervention, concerns which have also been reported for CHWs using malaria RDTs in Uganda [13].

While the use of teachers to deliver anthelmintics treatment in schools has been found to be acceptable elsewhere [25,34], their use in the delivery of IST in schools appeared to be generally unacceptable to most participants in this study. The main reason for their lack of acceptability is that IST involves taking blood samples from school children, something that is perceived to be beyond teachers’ scope of practice and can therefore create role conflicts, overburden the already overworked teachers and undermine their ability to discharge their normal duties. This finding is different from that reported by Magnussen *et al.* where the use of teachers to diagnose (based on temperature, rather than RDTs) and treat school children for malaria in Tanzania was found to be acceptable to both teachers and parents, and parents were not opposed to teachers taking blood samples from their children provided that they were supervised by health workers [26]. The minority of participants who supported the use of teachers to deliver the intervention in schools considered that the RDT screening process was a simple procedure that teachers, if well trained and supervised could deliver with the support of health workers, because of the existing trusting relationship that they have both with school children and the general community. While the testing caused concerns, the use of teachers to administer treatment to school children after testing was, however, acceptable to most participants as it reflected their previous experience with other school health programmes that involved providing treatment to school children without parasitologically confirmed diagnosis [27,28].

Most of the concerns raised about the IST intervention were related to rumours about blood sample taking and covert HIV testing. Rumours, particularly those about blood, are often directly related to medical research and health interventions and are very common across sub-Saharan Africa [6,35]. While such rumours may not be so much of a problem under routine implementation of health interventions, they can still exercise a motivational force among even those who do not necessarily believe them and can have serious consequences for the uptake of and adherence to these interventions [35]. In this study for instance, these rumours were reported to have affected recruitment of study participants, led to withdrawals and non-adherence to treatment. It was also reported that these rumours had affected the routine implementation of other health programmes delivered through schools such as vitamin A supplementation and tetanus toxoid campaigns. The existence of rumours reflects the nature of the relationships between those implementing and those receiving the intervention [36]. The findings from this study illustrate that these relationships are influenced by perceptions of professional roles and responsibilities, social relationships (us *vs* them) and experiences (CHWs *vs* teachers, teachers *vs* health workers, health workers *vs* CHWs). As such, the involvement of the local community accompanied by proper community sensitization may be one way of addressing people’s concerns about the intervention if it is to be implemented as a strategy for school-based malaria control.

## Conclusions

The findings from this study have a number of implications for policy and the future implementation of IST. In particular, although school-based malaria control through IST for malaria was widely acceptable to most stakeholders, improved community health education on the impact of asymptomatic parasitaemia is needed to help reinforce knowledge on the consequences of asymptomatic parasitaemia on the health and education of school children, because lack of such knowledge may undermine full adherence to treatment among children who are seemingly healthy. It was also clear that use of anti-malarial drugs with simpler regimens would help improve treatment adherence. In addition, proper community sensitization and involvement of local leaders is important in ensuring community ownership of the intervention and dispelling fears and myths about IST, particularly the screening component. In terms of who delivers IST, the general consensus of stakeholders was that health workers were best placed to undertake the screening and provide treatment, although some stakeholders felt that CHWs who were adequately trained and supervised could also implement the intervention. Most participants were opposed to teachers taking finger prick blood samples from children, but all recognized that the involvement of teachers will be critical to the success of the programme.

## Competing interests

The authors declare that they have no competing interests.

## Authors’ contributions

GO led the data collection, data analysis and developed the manuscript. SN and KEH were responsible for fieldwork supervision and contributed to the final manuscript. KH assisted in the interpretation of the results and commented on the manuscript. SB was responsible for the overall project management and contributed to the study design, interpretation and writing of the manuscript. CJ contributed to study design, interpretation and writing of the manuscript. All authors read and approved the final manuscript.
